# Identification and analysis of methylation call differences between bisulfite microarray and bisulfite sequencing data with statistical learning techniques

**DOI:** 10.1186/1471-2105-16-S3-A7

**Published:** 2015-02-13

**Authors:** Matthias Döring, Gilles Gasparoni, Jasmin Gries, Karl Nordström, Pavlo Lutsik, Jörn Walter, Nico Pfeifer

**Affiliations:** 1 Department of Computational Biology and Applied Algorithmics, Max Planck Institute for Informatics, Campus E1 4, 66123 Saarbrücken, Germany; 2 Department of Genetics/Epigenetics, Saarland University, Saarbrücken, Germany

## Background

DNA methylation is an epigenetic modification known to play a prime role in gene silencing and is an important topic in epigenetic research. However, due to technology-dependent errors there are inconsistencies between methylation measurements from different methods [1]. Incorrect methylation calls could result in the discovery of spurious associations between methylation patterns and specific phenotypes in epigenome-wide association studies (EWAS). We worked towards assigning a measure of confidence to individual CpGs to down-weigh or exclude positions with inconsistent measurements in such studies. We used methylation measurements from the Infinium HumanMethylation450 microarray (β450K) and whole genome bisulfite sequencing (βWGBS) to evaluate whether locus-specific measurement differences, Δβ = β450K − βWGBS, are predictable using statistical learning techniques.

## Methods

**Figure 1:**
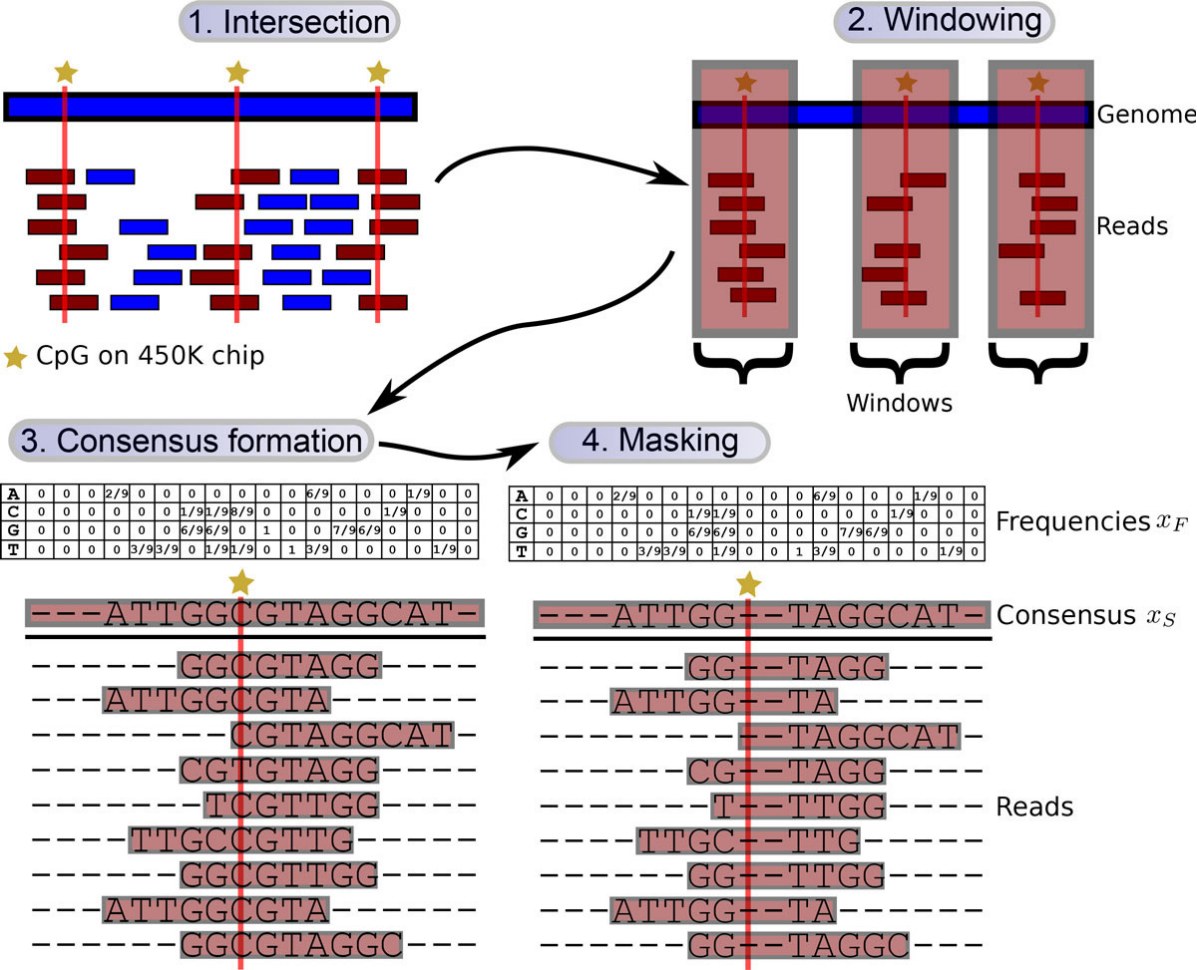
**Data preprocessing**. (1) Only reads overlapping with a CpG on the Infinium 450K chip are retained. (2) Windows are extended to the left and right of each CpG according to the maximum read length, yielding a uniform feature representation. (3) For each CpG, a consensus sequence is formed from its corresponding set of reads. Additionally, the position-specific frequency of each base is extracted. (4) Finally, CpG positions are masked by introducing gaps in the sequence or zeroing frequencies.

Methylation for Illumina WGBS data from HepaRGd7R2 was called with Bis-SNP [2], while methylation for Infinium 450K data from the same cell line was determined using RnBeads [3] and normalized with BMIQ [4]. For a uniform feature representation, we considered windows of reads overlapping with CpGs on the microarray (Figure 1). As predictors we examined sets of read sequences, their consensus sequences (with and without base frequencies), and non-sequence features such as base quality and depth of coverage. To obtain a predictive model independent of the methylation state, we masked CpG positions by introducing gaps or zeroing base frequencies.

To predict Δβ, we built support vector regression models based on Illumina WGBS data. Read similarity was measured with numerical, string [5-7], and set kernels [8]. We introduced the notion of hybrid string kernels to afford a similarity measure for both numeric and string input simultaneously. These kernels are based on scaling the motif similarity scores of two sequences according to the similarity of their base frequency profiles.

## Results

For a read-based set kernel utilizing the weighted degree kernel with shifts [6], we found that the predicted values of Δβ correlated significantly with the observed outcomes (r = 0.37, p-value < 2.2 · 10−16). Furthermore, the hybrid weighted degree kernel (r = 0.234) outperformed the weighted degree kernel with shifts (r = 0.22) by also considering the frequencies of individual bases in addition to the consensus sequences. Non-sequence features were less predictive of the outcome than the sequence, e.g., RBF kernels on base quality and depth of coverage attained only correlations of r = 0.057 and r = 0.003 with the outcome, respectively.

## Conclusion

To our knowledge, this is the first approach indicating that differences between methylation measurements from bisulfite sequencing and the Infinium HumanMethylation450 microarray are predictable from the reads. The results suggest that features beside the sequence play only a minuscule role in the emergence of inconsistent methylation measurements. We were able to show that, in this scenario, set kernels and hybrid string kernels provide well-suited similarity measures. Further work is necessary to validate the model's generalizability for data from other cell lines and to evaluate its practical merit.

## References

[B1] Dedeurwaerder S Defrance M Calonne C Denis H Sotiriou C Fuks F Evaluation of the Infinium Methylation 450K technology Epigenomics 2011 3 6 771 784 10.2217/epi.11.105 22126295

[B2] Liu Y Siegmund KD Laird PW Berman BP Bis-SNP: Combined DNA methylation and SNP calling for Bisulfite-seq data Genome Biol 2012 13 7 R61 10.1186/gb-2012-13-7-r61 22784381 PMC3491382

[B3] Assenov Y Müller F Lutsik P Walter J Lengauer T Bock C Comprehensive Analysis of DNA Methylation Data with RnBeads Nat Methods in press 10.1038/nmeth.3115 PMC4216143 25262207

[B4] Teschendorff AE A beta-mixture quantile normalization method for correcting probe design bias in Illumina Infinium 450K DNA methylation data Bioinformatics 2013 29 2 189 196 10.1093/bioinformatics/bts680 23175756 PMC3546795

[B5] Sonnenburg S Rätsch G Schäfer G Learning interpretable SVMs for biological sequence classification Research in Computational Molecular Biology 2005 Springer 389 407

[B6] Rätsch G Sonnenburg S Schölkopf B RASE: recognition of alternatively spliced exons in C. elegans Bioinformatics 2005 21 suppl 1 i369 i377 10.1093/bioinformatics/bti1053 15961480

[B7] Meinicke P Tech M Morgenstern B Merkl R Oligo kernels for datamining on biological sequences: a case study on prokaryotic translation initiation sites BMC Bioinformatics 2004 5 1 169 10.1186/1471-2105-5-169 15511290 PMC535353

[B8] Gärtner T Flach PA Kowalczyk A Smola AJ Multi-Instance Kernels Proceedings of 19th International Conference on Machine Learning 2002 San Mateo, CA: Morgan Kaufman 179 186 Edited by Sammut C, Hoffmann A

